# Molecular‐Level Control of the Intersheet Distance and Electronic Coupling between 2D Semiconducting and Metallic Nanosheets: Establishing Design Rules for High‐Performance Hybrid Photocatalysts

**DOI:** 10.1002/advs.202004530

**Published:** 2021-02-15

**Authors:** Tae‐Ha Gu, Xiaoyan Jin, So‐Jung Park, Min Gyu Kim, Seong‐Ju Hwang

**Affiliations:** ^1^ Department of Chemistry and Nanoscience College of Natural Sciences Ewha Womans University Seoul 03760 Republic of Korea; ^2^ Department of Materials Science and Engineering College of Engineering Yonsei University Seoul 03722 Republic of Korea; ^3^ Beamline Research Division Pohang Accelerator Laboratory (PAL) Pohang 37673 Republic of Korea

**Keywords:** charge reservoir, graphene, hybrid photocatalyst, photosensitizer, universal design rule

## Abstract

Hybridization with conductive nanospecies has attracted intense research interest as a general effective means to improve the photocatalytic functionalities of nanostructured materials. To establish universal design rules for high‐performance hybrid photocatalysts, correlations between versatile roles of conductive species and interfacial interaction between hybridized species are systematically investigated through fine‐control of intersheet distance between photocatalytically active TiO_2_ and metallic reduced graphene oxide (rGO)/RuO_2_ nanosheets. Molecular‐level tailoring of intersheet distance and electronic coupling between 2D nanosheets can be successfully achieved by restacking of colloidal nanosheet mixture with variable‐sized organic intercalants. While the shortest intersheet distance between restacked TiO_2_ and rGO nanosheets leads to the highest visible‐light‐driven photocatalytic activity, the best UV–vis photocatalyst performance occurs for moderate intersheet spacing. These results highlight the greater sensitivity of photoinduced electronic excitation to the intersheet distance than that of interfacial charge transfer. The rGO nanosheet can function as effective charge transport pathway and cocatalyst within ≈1.7 nm distance from the semiconducting nanosheet, and as efficient stabilizer for hybridized photocatalyst within ≈1.8 nm. The present study underscores that the intercalative restacking of colloidal nanosheet mixture with intercalants enables molecular‐level control of distance between 2D inorganic/graphene nanosheets, which provides a rational design strategy for high‐performance hybrid photocatalysts.

## Graphical abstract

Molecular‐level control of intersheet distance and electronic coupling between 2D graphene/inorganic nanosheets can be achieved by restacking colloidal nanosheet mixture with variable‐sized intercalants. The optimal conditions for the efficacy of conductive nanosheet as photosensitizer, electron reservoir, charge transport pathway, cocatalyst, and stabilizer can be established with restacked organic–semiconductor–conductor nanohybrids, providing novel rational design strategy for high‐performance hybrid photocatalysts.

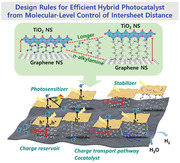

## 1 Introduction

Semiconductor‐assisted photocatalysis has received significant attention because of its effectiveness in converting solar energy into user‐friendly chemical energy.^[^ ^1^, ^2^ ^]^ The development of economically‐feasible high‐performance photocatalyst is indispensable for the industrial commercialization of solar energy‐based technologies such as the photosplitting of water into H_2_ and O_2_, the photoconversion of CO_2_ into valuable organics, and the environmental sanitization of harmful chemical species.^[^ ^2^, ^3^, ^4^ ^]^ To explore efficient photocatalysts, diverse methodologies have been exploited, such as chemical substitution, facet control, hybridization, surface modification, and defect formation.^[^ ^5^ ^]^ As one of the most effective synthetic strategies, hybridization with conductive nanospecies has been widely applied for a variety of photocatalyst materials.^[^ ^6^, ^7^, ^8^, ^9^ ^]^ The usefulness of this strategy relies on the versatile roles of hybridized conductor as photosensitizer, charge reservoir, charge transport pathway, cocatalyst, and stabilizer, enabling to optimize light absorption range, lifetime of charge carrier, charge transport properties, photocatalysis kinetics, and stability, respectively. Among the diverse conductive nanospecies reported to date, conductive 2D nanosheets (NSs) such as reduced graphene oxide (rGO) have been reported to exhibit a remarkably high efficiency as a hybridization matrix, as the nanometer‐level thickness and wide surface area of these 2D NSs enable strong interfacial electronic couplings with hybridized photocatalysts.^[^ ^7^ ^]^ The resulting enhanced electronic coupling with conductive 2D NSs provides an effective methodology for optimizing the photocatalytic functionalities of nanohybrids.^[^ ^1^ ^]^ To establish universal design rules for high‐performance hybrid photocatalysts, it is necessary to elucidate the optimal conditions for the versatile roles of the conductive species in hybrid materials and to understand the dominant factors governing the internal electron transfer processes between hybridized components.^[^ ^10^ ^]^ This goal requires the ability to precisely control the distance and coupling between the two different inorganic nanostructures. In contrast to plasmonic metal nanoparticles whose distance can be controlled via the surface binding of single‐strand DNA,^[^ ^11^, ^12^, ^13^ ^]^ there has been no efficient and economically feasible means of precisely controlling the distance and coupling between nanostructured inorganic compounds. Considering the high tunability of the interlayer spacings of intercalation compounds, layer‐by‐layer hybridization between a photocatalytically active semiconductor NS and a metallic conductor NS with various‐sized intercalants should allow the molecular‐level fine‐control of their intersheet distances and electronic couplings. The subtle tailoring of the interfacial electronic correlation between restacked semiconducting and metallic NSs can therefore offer valuable opportunities to determine the best conditions for optimizing the various roles of conductive NSs as photosensitizers, charge reservoirs, cocatalysts, and charge transport pathways. In addition, in situ spectroscopic analysis of these nanohybrids upon light illumination is useful for evaluating the efficiency of hybridized NSs as stabilization substrates depending on the intersheet distance. Based on the above points, restacked 2D semiconductor–conductor hybrid photocatalysts with molecularly‐tailored intersheet spacings are expected to be excellent model compounds for establishing design principles for efficient conductive NS‐based hybrid photocatalysts. Despite many studies devoted to 2D NS‐based hybrid photocatalysts being reported to date,^[^ ^6^, ^14^, ^15^, ^16^, ^17^ ^]^ at the time of this submission, we are unaware of any other systematic studies on the molecular‐level control of intersheet distances and interfacial electronic couplings between conductive NSs and photocatalytically active NSs to provide rational design rules for high‐performance hybrid‐type photocatalysts.

In this study, a series of layer‐by‐layer‐ordered nanohybrids of layered TiO_2_ NS and rGO NS with finely controlled intersheet distances are synthesized by restacking colloidal NSs with various *n*‐alkylamine/*n*‐alkyl alcohols as well as protons. The effects of the intersheet distance on the relative efficacy of rGO NSs as a photosensitizer, electron reservoir, charge transport pathway, cocatalyst, and stabilizer are systematically investigated to offer universal design principles for high‐performance rGO‐based hybrid materials. The chemical bonding nature and physicochemical properties of the present nanohybrids are also examined using several in situ/ex situ spectroscopy techniques to understand the underlying mechanism responsible for the evolution of their photocatalyst performances upon variation in the intersheet distance. The universal efficacy of intersheet distance control in tailoring the photocatalyst functionality of interstratified NS hybrid materials is further evidenced by another hybrid system composed of a semiconducting TiO_2_ NS and a metallic RuO_2_ NS.

## 2 Results and Discussion

### 2.1 Synthesis of 2D Restacked Inorganic/Reduced Graphene Oxide Nanohybrids with Molecular‐Level Control of Intersheet Distances

**Figure 1:**
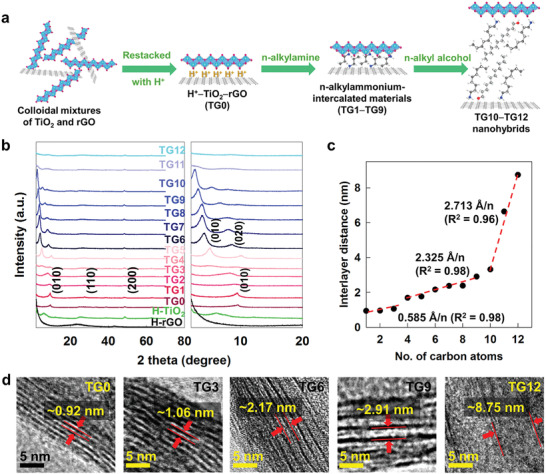
a) Schematic diagram of restacking route to *n*‐alkylamine‐intercalated TiO2–rGO nanohybrids. b) Powder XRD patterns, c) plot of interlayer distance versus carbon number, and d) cross‐sectional TEM images of TG*n* nanohybrids.

For the fine control of the intersheet distance between the layered TiO_2_ NSs and rGO NSs, the homogeneously restacked H^+^−TiO_2_−rGO nanohybrid was synthesized by the addition of protons into colloidal mixtures of 2D TiO_2_ and rGO NSs with a molar ratio of 5:1 (the obtained material is denoted as the TG0 nanohybrid). As depicted in **Figure** **1**a, *n*‐alkylammonium‐intercalated materials are prepared by reacting excess *n*‐alkylamine molecules with the TG0 nanohybrid (the obtained materials are denoted as TG*n* nanohybrids). For the synthesis of TG*n* nanohybrids with *n* = 10–12, the TG9 nanohybrid was reacted with *n*‐alkyl alcohol molecules, resulting in the cointercalation of alcohol molecules with additional basal expansion.^[^ ^18^ ^]^ As illustrated in Figure 1a, the control of the basal spacing of these TG*n* nanohybrids enables the tailoring of the intersheet distance between the TiO_2_ and rGO monolayers, since both the layered TiO_2_ and the rGO NSs are uniformly restacked in a layer‐by‐layer manner. According to powder X‐ray diffraction (XRD) analysis (Figure 1b; Figure S1, Supporting Information), all TG*n* nanohybrids commonly displayed well‐developed (0k0) peaks in the low‐angle region, indicating the formation of intercalative hybrid materials composed of layer‐by‐layer‐ordered NSs.^[^ ^18^ ^]^ As summarized in **Table** **1**, the intercalation of *n*‐alkylammine and/or *n*‐alkyl alcohol causes a gradual shift of (0k0) reflections toward low‐angle side with increasing length of the intercalant molecules.^[^ ^19^ ^]^ The lack of rGO‐related peaks confirms the homogeneous mixing of rGO with layered TiO_2_ NSs in the restacked lattice. The restacked TG*n* nanohybrids with *n* = 0–3 show a slight increase in interlayer distance (0.585 Å*n* ^−1^) with increasing carbon number (*n*) of the intercalant molecule, whereas an increase in the number of carbon atoms beyond *n* = 3 causes a steeper expansion of interlayer distance (2.325 Å *n* ^−1^) (Figure 1c). The additional reaction with n‐alkyl alcohol molecules with *n* ≥ 10 causes a greater increase in the interlayer distance (2.713 Å *n* ^−1^). The observed slope change in this plot reflects the alteration of the orientation of intercalated molecules in the interlayer space of TiO_2_/rGO lattice. As depicted in Figure S2, Supporting Information, the intercalated *n*‐alkylammonium ions are aligned parallel to the basal plane of the TiO_2_/rGO layers through host–guest electrostatic interactions in the region of *n* = 0–3, whereas the intercalated longer‐chain alkylammonium ions with *n* ≥ 4 have a tilted orientation with respect to the host layers owing to the guest–guest hydrophobic interactions.^[^ ^1^ ^]^ In addition to the (0k0) reflections, the distinct in‐plane (200) XRD peak is discernible at 2*θ* = ≈47°, confirming the maintenance of the layered TiO_2_ lattice. The present XRD results clearly demonstrate that the restacking process of colloidal NS mixtures with intercalant molecules provides an effective universal methodology for the fine‐tuning of the distance between inorganic nanostructures at the molecular level. This method is more economically feasible than the previously reported self‐assembly route of plasmonic metal nanoparticles with surface anchoring of single‐strand DNA.^[^ ^11^, ^12^, ^13^ ^]^ Subsequently, the molecular‐level control of the intersheet distance was clearly confirmed by transmission electron microscopy (TEM) observations, where the layer‐by‐layer‐ordering of TiO_2_/rGO NS with gradually increasing basal spacing was apparent (Figure 1d). Indeed, the intersheet distances of the nanohybrids determined by TEM are in good agreement with those estimated by powder XRD analysis. The porous stacking structures composed of 2D NSs with micrometer‐level lateral dimension were demonstrated in field emission‐scanning electron microscopy (FE‐SEM) images, see Figure S3, Supporting Information. The homogeneous intercalative hybridization between *n*‐alkylammonium ions and the TiO_2_/rGO NSs was confirmed by energy dispersive spectroscopy (EDS)‐elemental mapping analysis. As depicted in Figure S4, Supporting Information, the uniform distribution of the C, N, Ti, and O elements is discernible for the entire area of the nanohybrids. The quantity of intercalated organic species was determined by elemental analysis (Table 1).

**Table 1:** Structural parameters for TG*n* nanohybrids

Material	Interlayer distance [nm]	Length of guest [nm]	Quantity of guest [%]
TG0	0.92(2)	0.250	–
TG1	0.94(1)	0.417	10.3
TG2	0.96(1)	0.513	10.9
TG3	1.06(3)	0.648	11.4
TG4	1.69(2)	0.763	12.3
TG5	1.77(4)	0.898	12.5
TG6	2.17(6)	1.019	13.6
TG7	2.38(1)	1.168	14.5
TG8	2.39(4)	1.282	15.3
TG9	2.91(9)	1.320	15.9
TG10	3.32(4)	1.480	16.2
TG11	6.64(2)	1.580	20.7
TG12	8.75(1)	1.710	22.1

### 2.2 Electronic Structure

**Figure 2:**
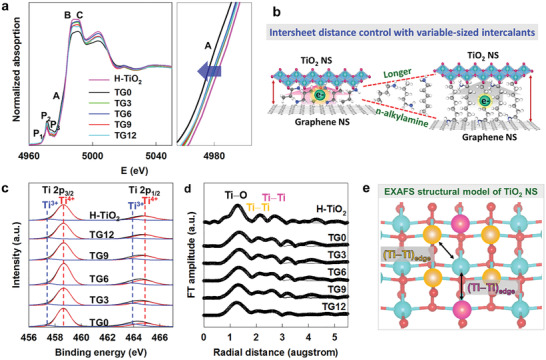
a) Ti K‐edge XANES spectra of TG*n* nanohybrids. b) Schematic diagram for fine‐control of intersheet distance and interfacial electronic coupling between TiO_2_ and rGO NSs. c) Ti 2p XPS, d) Ti K‐edge FT‐EXAFS, and e) EXAFS structural model of layered TiO_2_ NS.

The electronic configurations and local structures of the TG*n* nanohybrids were investigated using bulk‐sensitive X‐ray absorption spectroscopy (XAS) and surface‐sensitive X‐ray photoelectron spectroscopy (XPS) techniques to examine the effects of the intersheet distance on the interfacial chemical interaction and the electronic coupling between restacked TiO_2_ and rGO NSs. As presented in the Ti K‐edge X‐ray absorption near‐edge structure (XANES) of **Figure** **2**a, all materials under investigation exhibit three pre‐edge peaks (P_1_, P_2_, and P_3_) related to dipole‐forbidden 1s → 3d transitions, and three main‐edge features (A, B, and C) assigned to dipole‐allowed 1s → 4p transitions.^[^ ^18^, ^20^, ^21^ ^]^ The present TG*n* nanohybrids commonly display very similar spectral features to those of lepidocrocite‐type TiO_2_, indicating the maintenance of the original layered structure of the TiO_2_ NSs. In addition, the edge energies of the TG*n* nanohybrids are nearly identical to that of the reference TiO_2_, confirming the tetravalent Ti oxidation state of these materials. A closer inspection reveals that a shortening of the intersheet distance causes a slight shift of the edge energy toward the low‐energy side, indicating a reduction in the Ti oxidation state. Since the interfacial electronic coupling with electron‐rich rGO NSs induces electron injection into the TiO_2_ NSs (Figure 2b), the observed lowering of the edge energy can be interpreted as clear evidence for an enhanced charge transfer between neighboring TiO_2_ NSs and rGO NSs. The observed gradual change in the edge energy underscores the effectiveness of intersheet distance control with variable‐sized intercalants in tailoring the interfacial electronic coupling between the restacked semiconductor and conductor NSs, as illustrated in Figure 2b. The observed increase of the edge energy upon elongation of organic chain strongly suggests that the intercalated organic molecules act as spacers rather than as charge transferring species. Since the alkyl groups in organic chain molecule can act as electron donating agents to neighboring TiO_2_ NS, the TG12 with the longest organic chain molecule is expected to show the lowest Ti oxidation state due to the greater electron donating power of longer chain molecules. This expectation does not match with the XANES results, allowing to rule out the possible role of intercalated organic molecules as electron transferring species.

The evolution of interfacial charge transfer between the rGO NSs and TiO_2_ NSs upon variation in the intersheet distance was further probed by surface‐sensitive XPS analysis. As plotted in Figure 2c, the shortening of the intersheet distance enhances the low‐energy component of the Ti 2p XPS features, indicating that reduction of the surface Ti species took place.^[^ ^22^ ^]^ As summarized in Table S1, Supporting Information, peak convolution analysis clarifies the increase of the Ti^3+^/Ti^4+^ ratio with a decreasing intersheet distance, verifying the controllability of the interfacial charge transfer based on the present restacking strategy. The XANES and XPS results provide strong evidence for the fine control of the interfacial electronic coupling between restacked TiO_2_ and rGO NSs through variation in their interlayer distance, leading to modification of the Ti electronic structure both in the bulk and surface regions.

The local structural variation of TiO_2_ NS upon changing the intersheet distance was then quantitatively evaluated using Ti K‐edge extended X‐ray absorption fine structure (EXAFS) analysis. As presented in the Fourier‐transformed (FT) EXAFS spectra of Figure 2d, all TG*n* nanohybrids commonly display typical spectral features of the lepidocrocite‐type layered TiO_2_ phase. The three intense FT peaks at ≈1.3, ≈2.2, and ≈2.7 Å are assigned as (Ti—O) and two different edge‐shared (Ti—Ti) coordination shells, respectively (see Figure 2e).^[^ ^23^ ^]^ All EXAFS data can be well reproduced considering a lepidocrocite‐type TiO_2_ structure. According to the non‐linear curve fitting analysis, a shortening of the intersheet distance results in elongation of the (Ti—O) and (Ti—Ti) bond distances (Table S2, Supporting Information), confirming an enhanced reduction of the Ti oxidation state. Furthermore, the Debye–Waller (*σ* ^2^) factor becomes larger with an increase of the intersheet distance, reflecting the increase of local structural deformation of TiO_2_ layer. Such a structural disordering can be ascribed to the facile conformation change of intercalated long chain organic molecules due to their easy molecular rotation and kink formation. This finding strongly suggests the significant influence of the geometry of intercalated organic molecules on the local structure of TiO_2_ layer.^[^ ^24^ ^]^

### 2.3 Photocatalytic Activity

**Figure 3:**
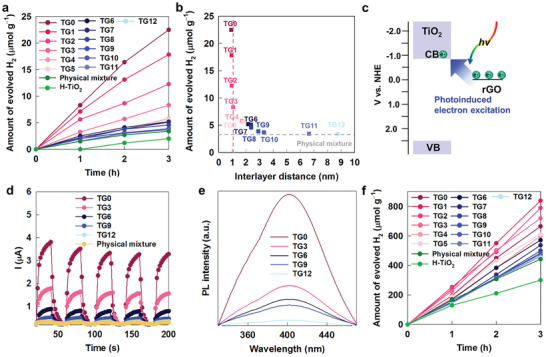
a) Photocatalytic activity for visible light‐induced H_2_ evolution and b) plot for amount of evolved H_2_ versus intersheet distance of TG*n* nanohybrids. c) Schematic model for photoinduced electronic excitation from electronic state of rGO to CB of TiO_2_. d) Visible light‐induced photocurrent generation, e) OH▪ radical formation, and f) UV–vis‐induced photocatalytic H_2_ evolution by TG*n* nanohybrids. All the data of photocatalytic activity tests were averaged over five independent measurements. The standard deviation ranged from 0.3 to 2 µmol g^−1^ for visible light‐induced H_2_ evolution and from 2 to 18 µmol g^−1^ for UV–vis‐induced H_2_ evolution.

The effect of the intersheet distance on the photocatalytic activities of the TG*n* nanohybrids was investigated by testing their visible light (*λ* > 420 nm)‐induced H_2_ generation. As depicted in **Figure** **3**a, all TG*n* nanohybrids show distinct photocatalytic activities for visible light‐induced H_2_ generation, which is in stark contrast to the pristine layered TiO_2_ displaying no significant photocatalytic functionality. Such an observation provides strong evidence for the efficient role of the rGO NSs as a photosensitizer to impart a visible light harvesting ability on the wide bandgap TiO_2_ NSs.^[^ ^25^ ^]^ Among the present nanohybrids, the highest photocatalytic activity was observed for TG0, which possesses the smallest intersheet distance. Indeed, an increase in the intersheet distance results in a gradual depression of the visible light photocatalytic activity, clearly demonstrating an inverse relationship between the photosensitizer efficiency of rGO and the intersheet distance with the TiO_2_ NSs. It is worthwhile to mention that the influence of organic composition change on the photocatalytic activities of TG*n* nanohybrids was also examined by normalizing the amounts of H_2_ molecules evolved with respect to the TiO_2_ content of these materials (Figure S5, Supporting Information). Such a normalization does not change the overall trends in the intersheet distance dependence of the photocatalytic activity of TG*n* nanohybrids. To examine the influence of the hydrophobicity of intercalated organic intercalants on the photocatalyst performance, the organic‐free hydrophilic nanohybrid was also synthesized as a reference by restacking of TiO_2_ and rGO NSs with Al_13_^7+^ Keggin nanoclusters and the photocatalytic activity of the obtained Al_13_^7+^ nanocluster‐intercalated TiO_2_–rGO nanohybrid (denoted as TG‐Keggin) was compared with that of hydrophobic organic‐intercalated TG nanohybrid. As presented in Figure S6a, Supporting Information, the obtained TG‐Keggin nanohybrid is found to show nearly identical intersheet distance to that of TG4 nanohybrid. The contact angle measurement confirms much lower hydrophobicity of TG‐Keggin nanohybrid than that of TG4, see Figure S6b, Supporting Information. Of prime importance is that, in spite of significant difference in surface hydrophobicity, both the hydrophilic TG‐Keggin and hydrophobic TG4 nanohybrids display nearly identical photocatalytic activity. This result clearly demonstrates that the photocatalyst performances of the restacked TiO_2_–rGO nanohybrids are mainly governed by intersheet distances not by hydrophobicity (Figure S6c, Supporting Information). Thus, the hydrophobicity of organic intercalant is concluded to have only a negligible effect on the photocatalytic activity of the TiO_2_–rGO nanohybrid. As plotted in Figure 3b, beyond the TG3 nanohybrid with a short intersheet distance of ≈1 nm, a further increase in the intersheet distance gives rise to a significant depression of the visible light photocatalytic activities for the TG4–TG12 nanohybrids, which is similar to that observed for a physical mixture of TiO_2_ and rGO NSs. This finding strongly suggests that the photosensitizer role of conductive rGO is fairly effective within the intersheet distance of ≤1 nm, and becomes notably weaker beyond this distance. Such a high sensitivity of the photosensitizer efficiency to the intersheet distance can be explained by the fact that the photosensitizer role relies on the direct photoinduced excitation from rGO to the conduction band (CB) of TiO_2_ through the electron tunneling (see Figure 3c).^[^ ^26^ ^]^ Since rGO can be photoexcited via the delocalization and energy excess dissipation, the electron transfer from rGO to TiO_2_ occurs with the extension of light absorption property.^[^ ^27^, ^28^ ^]^ Such an electron tunneling is reported to be valid within ≈1 nm and sensitively dependent on the distance change.^[^ ^29^, ^30^, ^31^ ^]^ This is in good agreement with the present results showing that the rGO can play an effective role of photosensitizer for the TG0−TG4 nanohybrids with smaller intersheet distance of ≈1 nm. In contrast, the visible light photocatalytic activities of the other nanohybrids with longer intersheet distance of >1 nm are nearly identical to that of physical mixture of TiO_2_ and rGO, confirming negligible electronic tunneling between these NSs at the elongated intersheet distance. The detrimental effect of intersheet expansion on the efficacy of rGO as a photosensitizer was further verified by measuring the photocurrent generated by visible light irradiation. As presented in Figure 3d, all TG*n* nanohybrids display a distinct photocatalytic performance for visible light‐induced photocurrent generation, confirming the photosensitizer role of the rGO NSs. The amount of photocurrent generated decreases with an increase in the intersheet distance between the TiO_2_ and rGO NSs. Among the various materials under investigation, the TG0 nanohybrid with the smallest intersheet distance can induce the most efficient photocurrent generation under visible light irradiation. The highest visible light photocatalytic activity of TG0 was cross‐confirmed by the most efficient generation of OH▪ radicals under visible light irradiation, as shown in Figure 3e.^[^ ^32^ ^]^ The increase in the intersheet distance results in a reduction in the hydroxyl radical concentration, therefore confirming the high sensitivity of the photosensitizer role of rGO to the intersheet distance with the photocatalyst TiO_2_ NSs.

The influence of the intersheet distance on the photocatalyst functionalities of the TG*n* nanohybrids was also examined with UV−vis irradiation. As plotted in Figure 3f, in the presence of hole scavenger, the photocatalyst performance of TiO_2_ for UV−vis‐induced H_2_ production is more effective for the TG1–TG3 nanohybrids than for the TG0 nanohybrid, emphasizing the higher efficacy of short‐chain organic molecules as intercalants compared to protons. Beyond TG3, a further expansion of the intersheet spacing results in a significant decrease in the photocatalytic activity. In addition, TG8–TG12 nanohybrids exhibited marginally better photocatalytic performances than that of the physical mixture of TiO_2_ and rGO NSs, indicating that the beneficial effect of heterolayered hybridization with conductive rGO NSs becomes notably weaker for an extended intersheet distance of ≥3 nm. Like visible light photocatalytic activity, the normalization of H_2_ generation with respect to the TiO_2_ composition leads to only a negligible change in the overall trends in the dependence of UV–vis photocatalyst performances of TG*n* nanohybrids on the intersheet distance, see Figure S4, Supporting Information. Of prime importance is the fact that the observed intersheet distance dependence of the UV–vis photocatalytic activity is quite different from that of the visible light photocatalytic activity, which exhibits a gradual reduction with an increase in the carbon number from 0 to 3. The observed non‐gradual dependence of the UV–vis photocatalytic activity on the intersheet distance cannot be explained by the role of rGO as a photosensitizer. It is therefore worth mentioning that the intercalated *n*‐alkylamine/*n*‐alkyl alcohol molecules in the TG*n* nanohybrids could act as hole scavengers to enhance H_2_ generation, since the UV‐induced generation of holes in the valence band (VB) of TiO_2_ can cause the decomposition of these interlayer organic species.^[^ ^33^ ^]^ This effect may account for the superior UV–vis photocatalyst performances of the TG1–TG3 compared to that of the organic‐free TG0 nanohybrid. To determine the possible role of the organic intercalant as a hole scavenger, the UV–vis photocatalytic activities of the TG*n* nanohybrids were also measured in the absence of a sacrificial agent. As presented in Figure S7, Supporting Information, in the absence of a sacrificial agent, no significant photocatalytic activity was observed for the organic‐intercalated TG1–TG3 nanohybrids. This result provides strong evidence for the negligible contribution of the organic intercalant as a sacrificial agent. This conclusion was cross‐confirmed by powder XRD analysis of the TG1–TG3 nanohybrids subjected to the photocatalytic test, which exhibited no marked change in the position of the (0k0) reflections (see Figure S8, Supporting Information). Conversely, the other TG6–TG12 nanohybrids containing longer organic intercalants displayed weak but distinct photocatalytic activities under UV–vis irradiation with no sacrificial agent, suggesting the possible role of the long‐chain molecule as a hole scavenger. However, these materials also retained their original XRD patterns after the photocatalytic tests (Figure S8, Supporting Information), strongly suggesting the marginal contribution of long‐chain organic molecules as hole scavengers. These results clearly demonstrate that the observed non‐gradual distance‐dependence of the UV–vis photocatalytic activity of the TG*n* nanohybrids cannot be ascribed to the role of the organic intercalant as a hole scavenger, but instead, it is an intrinsic property of the interstratified semiconductor–conductor NS hybrid system. The origin of the non‐gradual dependence of the UV–vis photocatalytic activity on the intersheet distance will be discussed later in Section 2.5, based on spectroscopic evidence. In addition, the structural evolution of TG nanohybrids upon the photocatalytic H_2_ evolution test with hole scavenger was also examined to verify the stability of intercalated organic molecules during the photoreaction. As plotted in Figure S9, Supporting Information, all the present TG nanohybrids do not show any marked change of XRD peaks after the photocatalyst tests, emphasizing the high photostability of intercalated organic species.

### 2.4 Effect of Intersheet Distance on the Role of Reduced Graphene Oxide Nanosheet as Photosensitizer

**Figure 4:**
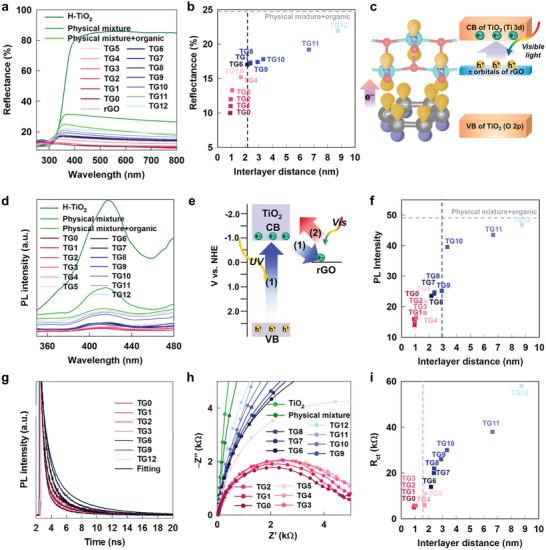
a) Diffuse reflectance UV−vis spectra and b) plot of reflectance versus intersheet distance of TG*n* nanohybrids. c) Schematic model for effect of orbital overlaps between adjacent rGO and TiO_2_ NSs on interfacial electronic excitation. d) PL spectra of TG*n* nanohybrids. e) Schematic model for interfacial charge transfer between restacked TiO_2_ and rGO NSs. f) Plot of PL intensity versus intersheet distance, g) TRPL spectra, h) Nyquist plots, and i) plot of *R*_ct_ versus intersheet distance of TG*n* nanohybrids.

The evolution of the photosensitizer efficiency of the rGO NSs upon variation in the intersheet distance was investigated using diffuse reflectance UV–vis spectroscopy. As presented in **Figure** **4**a, a distinct absorption edge corresponding to the bandgap energy appears for the TiO_2_ NSs, whereas the rGO NSs do not exhibit any distinct absorption edge, confirming the semiconducting and metallic nature of the TiO_2_ and rGO NSs, respectively. In addition, all TG*n* nanohybrids displayed much weaker reflectances than the TiO_2_ NSs, highlighting the contribution of the rGO NSs as a photosensitizer to enhance the visible light absorptivity.^[^ ^8^ ^]^ The visible light reflectance of the TG*n* nanohybrids was found to increase upon increasing the intersheet distance, reflecting a reduction in the photosensitizer efficacy of the rGO NSs. It is worthwhile to mention that the physical mixture of TiO_2_ and rGO NSs with the molar ratio of 5:1 shows weaker UV–vis light absorptivity than those of TG9–TG12, which is attributable to significant difference in the TiO_2_ and rGO contents between the organic‐free physical mixture and the organic‐intercalated TG9–TG12 nanohybrids. To rule out the interference effect of dissimilar composition of organic‐free physical mixture, another physical mixture of TiO_2_, rGO, and organic nonylammine with the same composition as TG9 was also prepared. This organic‐containing physical mixture exhibits almost identical UV–vis light absorptivity to those of TG9–TG12, reflecting only a weak electronic coupling of TiO_2_ and rGO NSs in TG9–TG12 nanohybrids. It is also noteworthy that the TG0–TG3 nanohybrids with short intersheet distances commonly show very weak visible light reflectance (i.e., a strong visible light absorptivity) similar to that of pure rGO, emphasizing the highly efficient photosensitizer role of rGO NSs in these nanohybrids. As shown in Figure 4b, the plot of visible light reflectance versus intersheet distance displays a marked slope change at around TG6, indicating the remarkable degradation of visible light absorptivity and a significant lowering of the photosensitizer efficiency of rGO at an extended distance of >2 nm. In addition to the increase in visible light absorption, restacking with rGO NS induces a weak but distinct red‐shift of the absorption edge, implying a reduction in the bandgap energy (*E*_g_) of TiO_2_ NSs. The observed lowering of *E*_g_ upon hybridization with the rGO NSs can be interpreted as a result of photoinduced electron excitation from the occupied level of the rGO NSs to the CB of the TiO_2_ NSs (Figure 3c).^[^ ^34^ ^]^ Since such a direct interfacial electronic excitation can be promoted by orbital overlaps between adjacent rGO and TiO_2_ NSs (Figure 4c), the photosensitizer efficiency of rGO is inversely proportional to the intersheet distance between the TiO_2_ and rGO NSs owing to the degradation of interfacial orbital overlap. The maximized photosensitizer efficiency at the shortest intersheet distance is responsible for the highest visible light photocatalytic activity of the TG0 nanohybrid.

### 2.5 Effect of Intersheet Distance on the Role of Reduced Graphene Oxide Nanosheet as Charge Reservoir

To probe the effect of the intersheet distance on the efficiency of rGO NSs as a charge reservoir, the charge recombination kinetics of the TG*n* nanohybrids were investigated using photoluminescence (PL) spectroscopy. As depicted in Figure 4d, all present TG*n* nanohybrids exhibited a significant decrease in their PL intensity upon hybridization with the rGO NSs, indicating a reduction in electron−hole recombination.^[^ ^7^ ^]^ The observed PL depression can be interpreted as a result of the effective transfer of photoexcited electrons in the CB of TiO_2_ to the empty level of neighboring rGO NSs (Figure 4e), resulting in the spatial separation of photoexcited electrons and holes. Such a depression of charge recombination is regarded as strong evidence for the effective role of the rGO NSs as a charge reservoir.^[^ ^34^ ^]^ As plotted in Figure 4f, the TG10–TG12 nanohybrids with elongated intersheet distances show comparable PL intensities to the organic‐containing physical mixture of TiO_2_ and rGO NSs, indicating the significant depression of the charge reservoir efficiency of rGO in these materials. An abrupt increase in the PL intensity beyond TG9 can be observed, indicating that the efficiency of rGO as a charge reservoir becomes notably weaker beyond ≈3 nm. This result indicates the weaker sensitivity of the charge reservoir role of rGO NS to the intersheet distance as compared with the photosensitizer role of rGO NS. Of prime importance is the fact that the PL intensities of TG1–TG3 are weaker than that of TG0, underscoring the fact that restacking with short‐chain organic molecules results in enhanced promotion of the charge separation between TiO_2_ and the rGO NSs than in the case of protons. As illustrated in Figure 4e, illumination of UV–vis light induces not only 1) the interfacial transfer of excited electrons from the CB of TiO_2_ to empty level of rGO, but also 2) the interfacial electronic excitation from the rGO to the CB of TiO_2_. Since the latter process 2) is a reverse process of the former process 1), the promotion of photoexcited electronic transition 2) depresses the role of rGO as a charge reservoir, resulting in an enhancement of the PL intensity. Considering the fact that the interfacial electronic excitation 2) becomes much more efficient at short intersheet distance than interfacial charge transfer 1), a less effective charge separation in TG0 than in TG1−TG3 can be interpreted as a result of the more efficient compensation of interfacial charge transfer 1) by reverse‐directional electronic excitation 2). The resulting superior charge‐reservoir role of rGO in the TG1–TG3 nanohybrids is mainly responsible for their higher UV–vis photocatalytic activities than that of TG0.

Additionally, the underlying mechanism for the interfacial charge transfer processes taking place in the TG*n* nanohybrids was probed with time‐resolved photoluminescence (TRPL) spectroscopy. As plotted in Figure 4g, the TG12 nanohybrid with the longest intersheet distance exhibited the slowest PL decay, indicating the depression of the charge transfer process with an increase in the intersheet distance. To determine the PL lifetimes of several decay pathways, all spectra were fitted using the triexponential function of the time‐dependent decay of PL intensity *I*(*t*):

(1) $$I\left(t\right)=A_{1}\exp\left(-t/\tau_{1}\right)+A_{2}\exp\left(-t/\tau_{2}\right)+A_{3}\exp\left(-t/\tau_{3}\right)$$

where *A*_i_ and *τ*_i_ are the relative weight and lifetime of the *i*th decay component. Based on the time domain of each component listed in Table S3, Supporting Information, the fast components (*τ*_1_, *τ*_2_) were assigned as non‐radiative decay pathways, whereas the radiative decay pathway was considered responsible for the slow component (*τ*_3_).^[^ ^34^ ^]^ Based on the relative weights of the three components, the first component shows an overwhelming major contribution to the overall time‐dependent PL decay. Although elongation of the intersheet distance increases the lifetimes of the fast components (*τ*_1_, *τ*_2_), the slow component exhibits longer lifetimes (*τ*_3_) for shorter intersheet distance. Since the lifetimes of fast components (*τ*_1_, *τ*_2_) are related to the efficiency of interfacial electron transfer from the TiO_2_ NS to rGO NS (i.e., the process 1) in Figure 4e),^[^ ^35^ ^]^ the increase in *τ*_1_ and *τ*_2_ upon elongation of the intersheet distance clearly demonstrates the depression of interfacial electron transfer between the restacked NSs. It should be noted that, among the TG0–TG3 nanohybrids, TG1 shows the shortest lifetime (*τ*_1_), which is in good agreement with its lowest PL intensity (Figure 4d). In contrast to the fast components, the slow component (*τ*_3_) corresponds to the radiative decay related to the band edge emission of TiO_2_.^[^ ^36^ ^]^ Among the various materials under investigation, the TG0 nanohybrid with the shortest intersheet distance exhibited the longest *τ*_3_ lifetime (Table S3, Supporting Information). This observation can be understood as a result of the enhancement of photoinduced electron excitation from rGO to TiO_2_ (i.e., process 2) in Figure 4e) with a decreasing the intersheet distance. The resulting enhanced electron injection from rGO to the CB of TiO_2_ leads to a prominent elongation of the lifetime (*τ*_3_) of radiative decay for the TG0 nanohybrid with the shortest intersheet distance, as shown in Figure 4g.

### 2.6 Effect of Intersheet Distance on the Role of Reduced Graphene Oxide Nanosheet as Charge Transport Pathway and Cocatalyst

The relative efficacy of the rGO NSs as a charge transport pathway in the nanohybrids was investigated using electrochemical impedance spectroscopy (EIS). As presented in Figure 4h, all TG*n* nanohybrids display similar Nyquist plots consisting of semicircles in the high‐ frequency region, in which the diameter of the semicircle becomes smaller upon hybridization with rGO. Because the diameter of the semicircle is inversely proportional to the charge transfer resistance (*R*_ct_) between the active material and electrolyte,^[^ ^37^ ^]^ the observed smaller semicircle diameters of the TG*n* nanohybrids compared to that of the TiO_2_ NSs emphasize the effective role of rGO as a charge transport pathway. The shortening of the intersheet distance decreases the radius of the semicircle, clearly demonstrating an improvement of the charge transfer kinetics at short intersheet distances. Of prime importance is the fact that the TG0–TG4 nanohybrids commonly display nearly identical semicircle radii, reflecting the similar charge transport kinetics of these materials. According to the fitting analysis carried out with an equivalent circuit (Figure 4i), the R_ct_ value remains almost identical for the TG0–TG4 nanohybrids, highlighting the high efficiency of rGO as a charge transport pathway within ≈1.7 nm. Beyond TG4, a further increase in the intersheet distance results in a gradual increase of *R*_ct_, clearly demonstrating that the role of rGO as a charge transport pathway becomes significantly depressed at distances greater than ≈1.7 nm.

In addition to playing the roles of a photosensitizer, charge reservoir, and charge transport pathway, the conductive rGO NSs are also known to play the role of a cocatalyst.^[^ ^38^ ^]^ Due to the fact that all TG*n* nanohybrids contain rGO NSs as an identical cocatalyst component, the cocatalyst functionality of rGO in these materials was determined by the efficiency of electron transport from the photocatalytically active TiO_2_ NS to the rGO cocatalyst.^[^ ^8^ ^]^ Thus, the order of the *R*_ct_ values of the TG*n* nanohybrids estimated from the EIS analysis also reflects the relative cocatalyst efficiency of rGO in these materials. The present EIS results strongly suggest that the rGO NSs can function as an effective cocatalyst within ≈1.7 nm, and that the cocatalyst efficiency significantly degrades beyond this distance. However, the observed similar efficiencies of rGO as the charge transport pathway and cocatalyst in the TG0–TG4 nanohybrids do not match the relative order of their visible photocatalytic activities, which show a marked gradual depression upon increasing the intersheet distance. This result strongly suggests the minor contributions of the rGO NSs as a charge transport pathway and cocatalyst to the improved visible‐light photocatalyst performance of the TG*n* nanohybrids.

### 2.7 Effect of Intersheet Distance on the Role of Reduced Graphene Oxide Nanosheet as Stabilizer

**Figure 5:**
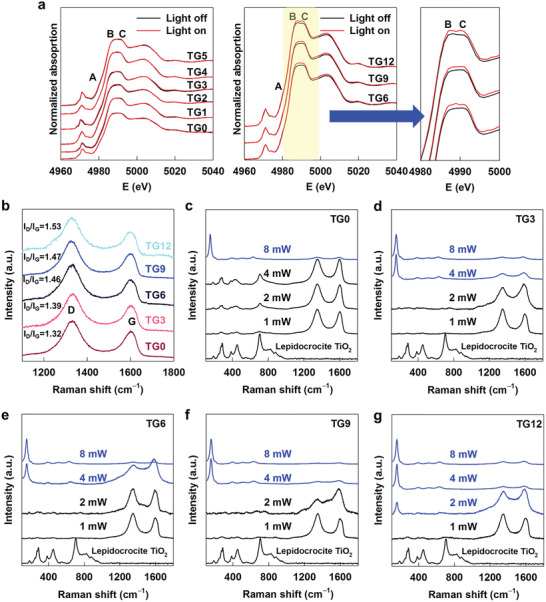
a) In situ Ti K‐edge XANES spectra under visible light irradiation and b) micro‐Raman spectra of TG*n* nanohybrids. Laser power‐dependent micro‐Raman spectra of c) TG0, d) TG3, e) TG6, f) TG9, and g) TG12.

The photoinduced changes of the local crystal and electronic structures of the TiO_2_ NS under visible light illumination were subsequently investigated by in situ Ti K‐edge XANES analysis for the TG*n* nanohybrids to examine the stabilizer role of the rGO NSs depending on the intersheet distance. As illustrated in **Figure** **5**a, the TG0–TG5 nanohybrids with shorter intersheet distances commonly exhibit only negligible spectral modifications under visible light irradiation, indicating no significant modification in the crystal and electronic structures of the TiO_2_ NSs. This result clearly demonstrates that the rGO NSs can function as an effective stabilization substrate for enhancing the photostability of the TiO_2_ NSs at a short intersheet distance of ≈1.8 nm. Conversely, illumination with visible light gives rise to a slight but distinct enhancement in main‐edge peaks B and C for the TG6–TG12 nanohybrids with longer intersheet distances, strongly suggesting the occurrence of photoinduced local structural frustration. Since the intensity of the main edge features is proportional to the density of unoccupied Ti 4p states,^[^ ^20^ ^]^ the observed enhancement of these peaks can be interpreted as a result of the weakened of (Ti—O) bond covalency under visible light irradiation, which leads to an increase of the hole density in the Ti ions.^[^ ^20^ ^]^ Such a decrease of the (Ti—O) bond covalency can be interpreted to be as a result of the photoinduced structural frustration of the layered TiO_2_ lattice, leading to a reduction in orbital overlaps between the Ti and O ion. The in situ XANES results presented here clearly demonstrate that the stabilizer role of the rGO NSs is effective within an intersheet distance of ≈1.8 nm, and becomes depressed as the intersheet distance increases further.^[^ ^24^ ^]^

The chemical bonding natures of the TG*n* nanohybrids were also examined by micro‐Raman spectroscopic analysis to verify the role of the rGO NSs as a stabilizer for the TiO_2_ NSs.^[^ ^39^, ^40^ ^]^ As shown in Figure 5b, all TG*n* nanohybrids display two intense peaks at ≈1360 and ≈1600 cm^−1^, which were assigned to the D and G bands of the graphene species, respectively, thereby confirming the incorporation of the rGO NSs in these materials.^[^ ^41^ ^]^ It was found that a decrease in the intersheet distance gives rise to a gradual lowering of the *I*_D_/*I*_G_ intensity ratio. Since a variation of the *I*_D_/*I*_G_ intensity ratio offers a sensitive indicator for the degree of structural disorder of graphene,^[^ ^42^ ^]^ the observed decrease in this ratio upon shortening of the intersheet distance clearly demonstrates a depression of the structural disorder of the rGO NSs. This result can be regarded as evidence for an enhancement of the chemical interactions between the rGO and TiO_2_ NSs upon reducing the intersheet distance, ultimately resulting in the prevention of the structural disorder in rGO.

Furthermore, the variations in the micro‐Raman spectra of the TG*n* nanohybrids were monitored with increasing laser power to verify the stabilizer role of the rGO NSs for the hybridized TiO_2_ NSs.^[^ ^34^, ^35^ ^]^ As plotted in Figure 5c, the TG0 nanohybrid retains the characteristic phonon lines of the lepidocrocite‐type TiO_2_ lattice in the low wavenumber region up to a laser power of 4 mW.^[^ ^43^ ^]^ A further increase in the laser power to 8 mW causes a significant spectral change to anatase TiO_2_‐type phonon lines, indicating the occurrence of the thermally‐induced phase transformation of the TiO_2_ NSs by the high‐power laser.^[^ ^44^ ^]^ Such a phase transition from lepidocrocite to anatase TiO_2_ occurs at a lower laser power of 4 mW for TG3–TG9 nanohybrids, whereas the TG12 nanohybrid with the longest intersheet distance experiences this phase transformation at a lower laser power of 2 mW. The increase in laser power required to induce this phase transition upon shortening of the intersheet distance provides strong evidence for an improvement of the stabilizer role of the rGO NSs, as evidenced by in situ XANES analysis.

To verify the universal efficacy of intersheet distance control in tailoring the visible and UV–vis photocatalytic activities of interstratified NS hybrid materials, an additional heterolayered 2D NS hybrid system composed of layer‐by‐layer‐ordered *n*‐alkylammonium–TiO_2_–RuO_2_ nanohybrids was synthesized by restacking of the photocatalytically active TiO_2_ NSs and metallic RuO_2_ NSs with organic intercalants (the obtained materials are denoted as TR*n* nanohybrids), as shown in Figure S10, Supporting Information. As observed in the TG*n* nanohybrids, alteration of the intercalant size is effective in finely controlling the intersheet distance between the restacked TiO_2_ and the RuO_2_ NSs. Similar to the case of the TG*n* nanohybrids, the TR*n* nanohybrids also display the unique dissimilar dependences of the visible and UV–vis photocatalyst functionalities on the intersheet distance (Figure S10, Supporting Information); the highest visible light‐driven photocatalytic activity was obtained for the TR0 nanohybrid with the shortest intersheet distance between the restacked TiO_2_ and RuO_2_ NSs, whereas the TR3 nanohybrid with a moderate intersheet distance exhibited the best UV–vis photocatalyst performance. This finding indicates that the unique correlations between the photocatalyst performance and the interfacial electronic coupling found in the present TG*n* hybrid system are universal for 2D conductive NS‐based hybrid photocatalysts.

## 3 Conclusion

**Figure 6:**
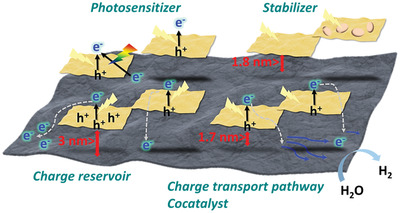
Rational design rules for exploring highly efficient hybrid photocatalysts with the fine‐control of interfacial distance.

In conclusion, the systematic investigation for restacked TG*n* nanohybrids with molecularly‐controlled intersheet distances provides valuable information regarding the conditions required for optimization of the photocatalytic performances of 2D NS‐based nanohybrids. The restacking of TiO_2_ and rGO NSs with *n*‐alkylamine/*n*‐alkyl alcohol molecules enabled molecular‐level control of their intersheet distances and interfacial electronic couplings. While the highest visible light photocatalytic activity was achieved for the TG0 nanohybrid with the shortest intersheet distance, moderate intersheet spacing was found to be favorable for optimizing the UV–vis photocatalytic activities of the restacked TG*n* nanohybrids. According to the systematic investigation for the optical and electronic properties of the TG*n* nanohybrids, the rGO NSs can play an effective photosensitizer role within an intersheet distance of ≤1 nm whereas the function of the rGO NSs as a charge reservoir shows a high level of effectiveness within ≈3 nm. The functions of rGO as a charge transport pathway and a cocatalyst were optimized within an intersheet distance of ≈1.7 nm and its role as an effective stabilization substrate within an intersheet distance of ≈1.8 nm. Based on the present experimental findings, the fine control of the distance between the photocatalyst and the photosensitizer/charge reservoir/charge transport pathway/cocatalyst/stabilizer can provide new rational design rules for exploring highly efficient hybrid photocatalysts with optimized hybridization effects. As illustrated in **Figure** **6**, the intimate surface contacts between the photocatalytically active component and the photosensitizer and the moderately spaced configuration with a charge‐transport‐pathway/cocatalyst (<1.7 nm), stabilizer (<1.8 nm), and charge reservoir (<3 nm) are expected to be effective in achieving high‐performance hybrid photocatalysts via optimization of the light absorption range, charge transport kinetics, charge carrier lifetime, and photocatalysis kinetics. Of prime importance is that the present exfoliation–restacking route provides a universal and economically feasible methodology to finely control the spacing and chemical/electronic interactions of a variety of 2D nanostructured materials. Considering the fact that hybridization with conductive 2D NSs is also useful in improving the electrocatalyst performances of nanostructured materials,^[^ ^45^ ^]^ the present synthetic strategy with the fine control of intersheet distances can be employed for the rational design and synthesis of high‐performance hybrid electrocatalysts. Currently, we are working on the application of the present synthetic strategy in diverse couples of functional inorganic NSs, including electrocatalyst/electrode NSs, to explore novel synergistically‐coupled high‐performance hybrid materials.

## 4 Experimental Section

##### Sample Preparation

The exfoliated layered TiO_2_ NS was synthesized in the form of colloidal suspension by the reaction of protonated layered titanate with 5 equivalent of tetrabutylammonium (TBA^+^) ions for >10 days.^[^ ^46^ ^]^ The aqueous colloidal suspension of exfoliated rGO NS was obtained by the reduction of graphene oxide (GO) synthesized via the modified Hummers’ method.^[^ ^47^ ^]^ The colloidal suspensions of layered TiO_2_ and rGO NSs commonly displayed zeta potentials of −45 and −37 mV, respectively, showing their negative surface charges (Figure S11, Supporting Information). The colloidal suspensions of 2D TiO_2_ and rGO NSs were mixed in the molar ratio of 5:1, yielding the stable colloidal mixtures of both the NSs. The addition of 1 m HCl solution into the obtained colloidal mixtures resulted in the synthesis of homogeneously restacked TG*n* nanohybrids. The simultaneous restacking of TiO_2_ and rGO NSs indicated the homogeneous incorporation of these nanoscale‐mixed NSs into the precipitated nanohybrid. The excess n‐alkylamine molecules (2.5wt% *n*‐alkylamine solution, 80 mL) were reacted with the obtained TG0 nanohybrid (80 mg) for 3 days to synthesize TG*n* nanohybrids. For the additional expansion of lattice spacing with *n* ≥ 10, *n*‐alkylammonium–TiO_2_–rGO nanohybrids (80 mg) were reacted with *n*‐alkyl alcohol molecules (2.5wt% *n*‐alkyl alcohol in 80 mL of distilled water/ethanol (1/1, v/v) mixed solution) for 5 days. After the reaction, the precipitated nanohybrids were restored by centrifugation, washed with distilled water and ethanol, and then dried in vacuum oven at 40 °C. Another hybrid system of the restacked TR*n* nanohybrids was synthesized in the same procedure as TG*n* nanohybrids, namely, the restacking of the colloidal mixture of exfoliated TiO_2_ and RuO_2_ NSs with intercalant species. The precursor RuO_2_ NS was obtained by the exfoliation reaction of protonated Na_0.2_RuO_2_ material with excess TBA^+^ ions for >10 days, as reported previously.^[^ ^48^ ^]^

##### Sample Characterization

The structural evolutions of the present nanohybrids upon the change of intercalant species were probed with powder XRD technique (Rigaku, *λ* = 1.5418 Å, 25 °C). TEM (Jeol JEM‐2100F, 200 kV) and FE‐SEM (Jeol JSM‐6700F microscope) were applied for probing the stacking structures of the present nanohybrids. The elemental compositions of the present nanohybrids were examined by monitoring EDS‐elemental maps with field emission‐scanning electron microscope (Jeol JSM‐6700F). The contact angle measurements were carried for water droplet with a DSA 100 (KRÜSS) instrument. To study the effect of intercalative hybridization on the electronic and local crystal structures of hybridized TiO_2_ NS, Ti K‐edge XAS data of the restacked nanohybrids were collected in a transmission mode at the beam line 10C of Pohang Accelerator Laboratory (PAL, Pohang, Korea). The energy calibration of all the present XAS data was done by simultaneously measuring the spectrum of Ti metal. The evolution of the chemical bonding nature of TG*n* nanohybrid under the irradiation of visible light was investigated with in situ Ti K‐edge XANES spectroscopic analysis. For measuring the in situ XANES spectra, the identical experimental setup was employed with the illumination of visible light from Xe lamp (300 W, Newport). The XPS data of the present nanohybrids were collected by XPS machine (Thermo VG, UK, Al K*α*). The measured XPS data were energy‐referenced with respect to the adventitious Au 4f peak at 84.0 eV to correct the interference by charging effect. To determine the optical properties of restacked nanohybrids, diffuse reflectance UV–vis spectra were collected with a JASCO V‐760 spectrometer. The PL spectra of the present nanohybrids were measured with a PerkinElmer FL 8500 at the excitation wavelength of *λ* = 320 nm to probe the charge recombination behavior of the present nanohybrids. The lifetimes of PL components in the present nanohybrids were evaluated with TRPL spectroscopy (MicroTime‐200) to study the effect of intersheet distance on the interfacial electron transfer between rGO and TiO_2_ NSs. The chemical bonding nature of TiO_2_ and rGO components in the present nanohybrids was studied with micro‐Raman spectroscopy using Horiba Jobin‐Yvon LabRam Aramis spectrometer, in which Ar ion laser (*λ* = 514.5 nm) was utilized as the excitation source. To probe the effect of intercalant size on the chemical stability of nanohybrids, micro‐Raman spectra were monitored with increasing laser power. The zeta potentials of colloidal TiO_2_ and rGO NSs were determined with Zetasizer Nano ZS (Malvern Instruments).

##### Photocatalytic Activity Test

The photocatalyst performances of the restacked nanohybrids were examined for the H_2_ evolution under the illumination of visible light and UV–vis. For the tests of visible light photocatalytic activity, both the optical cut‐off filter (*λ* > 420 nm) and water filter were used to remove UV and IR components from Xe lamp (300 W, Newport). As a sacrificial agent for hole scavenging, the mixed solution (100 mL) of 0.1 m Na_2_S and 0.02 m Na_2_SO_3_ was used for 50 mg of the photocatalyst. The amount of evolved H_2_ gas was evaluated with gas chromatography (Shimadzu GC‐2014). To check out the possible influence of the hydrophobicity of intercalated organic molecules on the photocatalytic activity of TG*n* nanohybrids, the alteration of sample dispersion ability upon the elongation of organic intercalant was examined prior to the photocatalyst test. As presented in Figure S12, Supporting Information, all the TG*n* samples exhibited good dispersion ability to form the homogeneous suspension for photocatalyst test. Even the TG12 nanohybrid with the longest organic chain molecule can be well‐dispersed with an assistance of magnetic stirring for few minutes. Additionally, the photocurrent generated by the present nanohybrids was measured using a three‐electrode cell, in which a Pt wire and a saturated calomel electrode electrode were employed as a counter electrode and a reference electrode, respectively. The coating of Nafion/ethanol/nanohybrids on indium tin oxide glass yielded the working electrode with the effective area of 1 cm^2^. Prior to the measurement, 0.1 m Na_2_SO_4_ electrolyte was purged with N_2_ bubbling for 0.5 h. For the generation of OH▪ radical, 10 mg of photocatalyst was reacted in 30 mL aqueous solution containing 3 mm terephthalic acid under the illumination of visible light. The formation of OH▪ radical was measured monitoring the fluorescence emission spectrum of 2‐hydroxyterephthalic acid. The EIS were measured using an impedance analyzer (IVIUM) with an AC voltage amplitude of 10 mV and a frequency range of 100 mHz–10 KHz.

##### Statistical Analysis

All the photocatalytic activity results were averaged over five independent measurements. The standard deviation ranged from 0.3 to 2 µmol g^−1^ for visible light‐induced H_2_ evolution and from 2 to 18 µmol g^−1^ for UV–vis‐induced H_2_ evolution.

## Conflict of Interest

The authors declare no conflict of interest.

## Supporting information

Supporting Information

## Data Availability

Research data are not shared.
