# Supplementary material for: Molecular‐Level Control of the Intersheet Distance and Electronic Coupling between 2D Semiconducting and Metallic Nanosheets: Establishing Design Rules for High‐Performance Hybrid Photocatalysts
Source: Adv Sci (Weinh). 2021 Feb 15;8(7):2004530. doi: 10.1002/advs.202004530 (PMC8024993; doi:10.1002/advs.202004530)
Supplement: Supplementary file 1 — Supporting Information [file ADVS-8-2004530-s001.pdf]

© 2021 Wiley-VCH GmbH

ADVANCED SCIENCE
Open Access

Supporting Information

for *Adv. Sci.*, DOI: 10.1002/advs.202004530

Molecular-Level Control of the Intersheet Distance and Electronic Coupling between 2D Semiconducting and Metallic Nanosheets: Establishing Design Rules for High-Performance Hybrid Photocatalysts

*Tae-Ha Gu, Xiaoyan Jin, So-Jung Park, Min Gyu Kim, and Seong-Ju Hwang**

Supporting Information

Molecular-Level Control of the Intersheet Distance and Electronic Coupling between 2D Semiconducting and Metallic Nanosheets: Establishing Design Rules for High-Performance Hybrid Photocatalysts

*Tae-Ha Gu, Xiaoyan Jin, So-Jung Park, Min Gyu Kim, and Seong-Ju Hwang**

T.-H. Gu, Prof. S.-J. Park
Department of Chemistry and Nanoscience, College of Natural Sciences, Ewha Womans University, Seoul 03760, Republic of Korea

Dr. X. Jin, Prof. S.-J. Hwang
Department of Materials Science and Engineering, College of Engineering, Yonsei University, Seoul 03722, Republic of Korea
E-mail: hwangsju@yonsei.ac.kr

Dr. M. G. Kim
Beamline Research Division, Pohang Accelerator Laboratory (PAL),
Pohang 37673, Republic of Korea

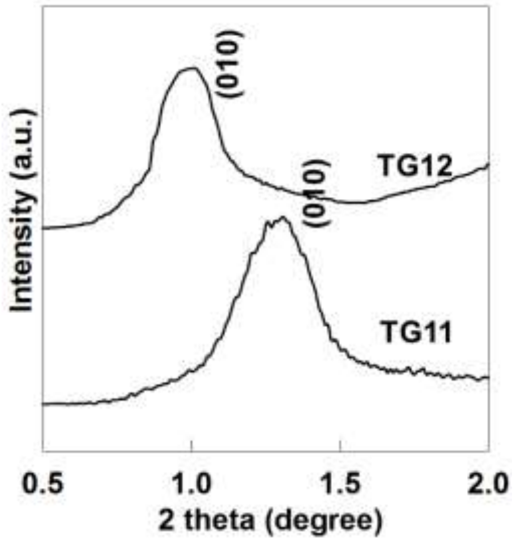

Figure S1. Small angle X-ray scattering (SAXS) patterns of TG11 and TG12 nanohybrids.

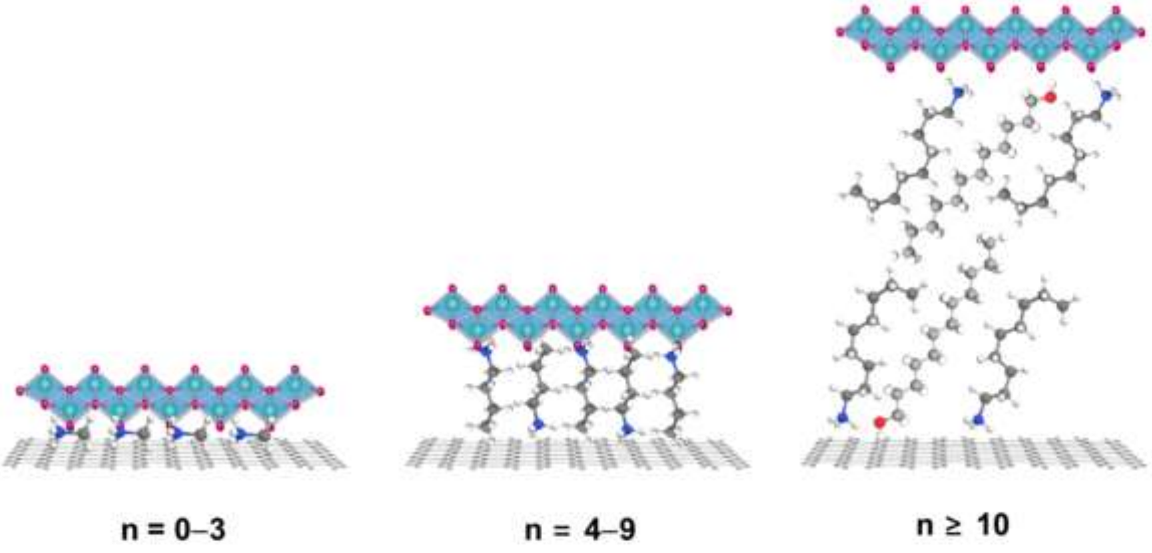

Figure S2. Schematic diagram for intercalated n-alkylammonium/n-alkyl alcohol molecules of TGn nanohybrids.

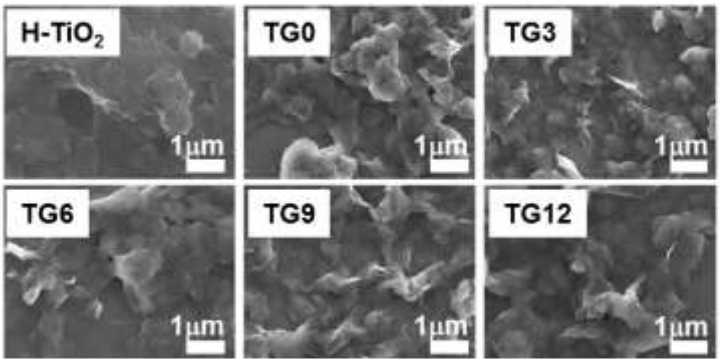

Figure S3. Field emission-scanning electron microscopy (FE-SEM) images of TGn nanohybrids.

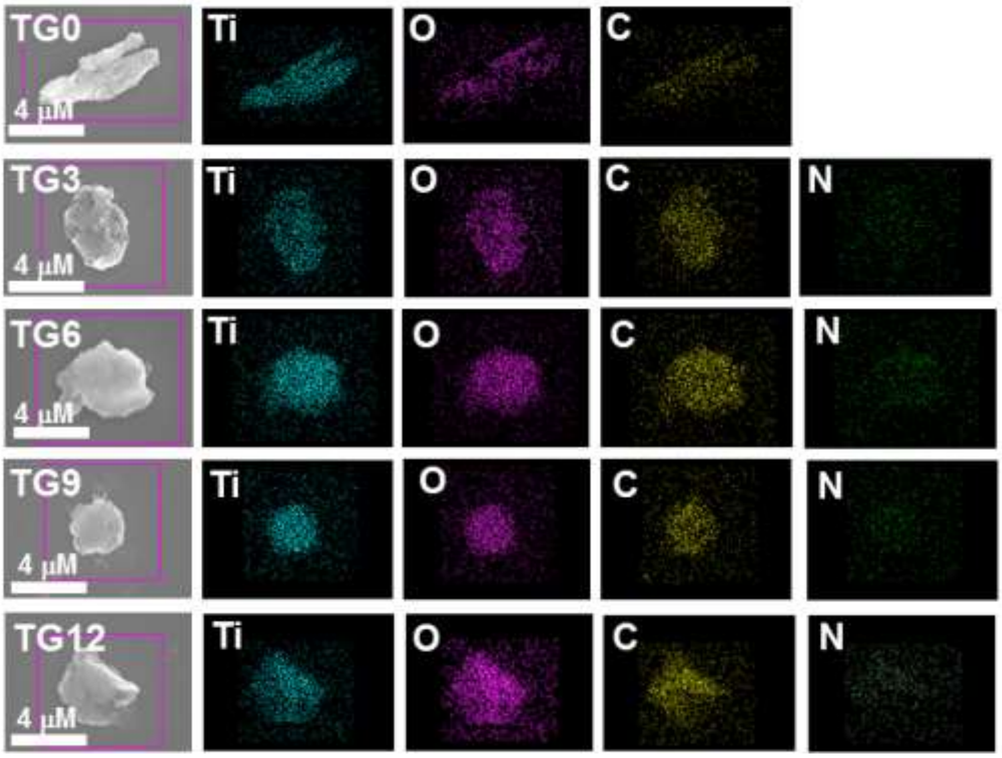

Figure S4. Energy dispersive spectrometry (EDS)–elemental maps of TGn nanohybrids.

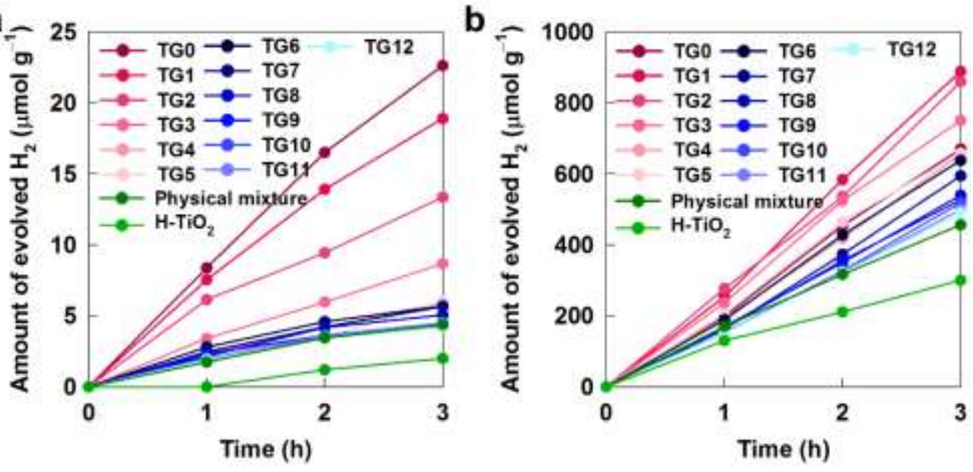

Figure S5. Photocatalytic activities of TGn nanohybrids for (a) visible light-induced and (b) UV−visible-induced H_2 evolution normalized by their TiO_2 contents.

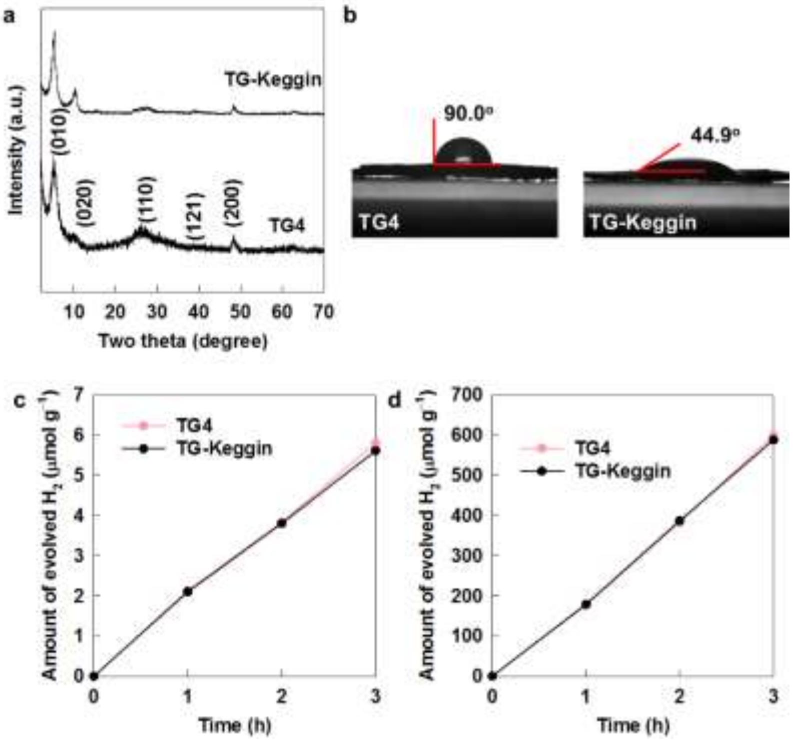

Figure S6. (a) Powder X-ray diffraction (XRD) patterns and (b) contact angle images of TG-Keggin and TG4 nanohybrids. Photocatalytic activities of TG-Keggin and TG4 nanohybrids for (c) visible light-induced and (d) UV–visible-induced H_2 evolution.

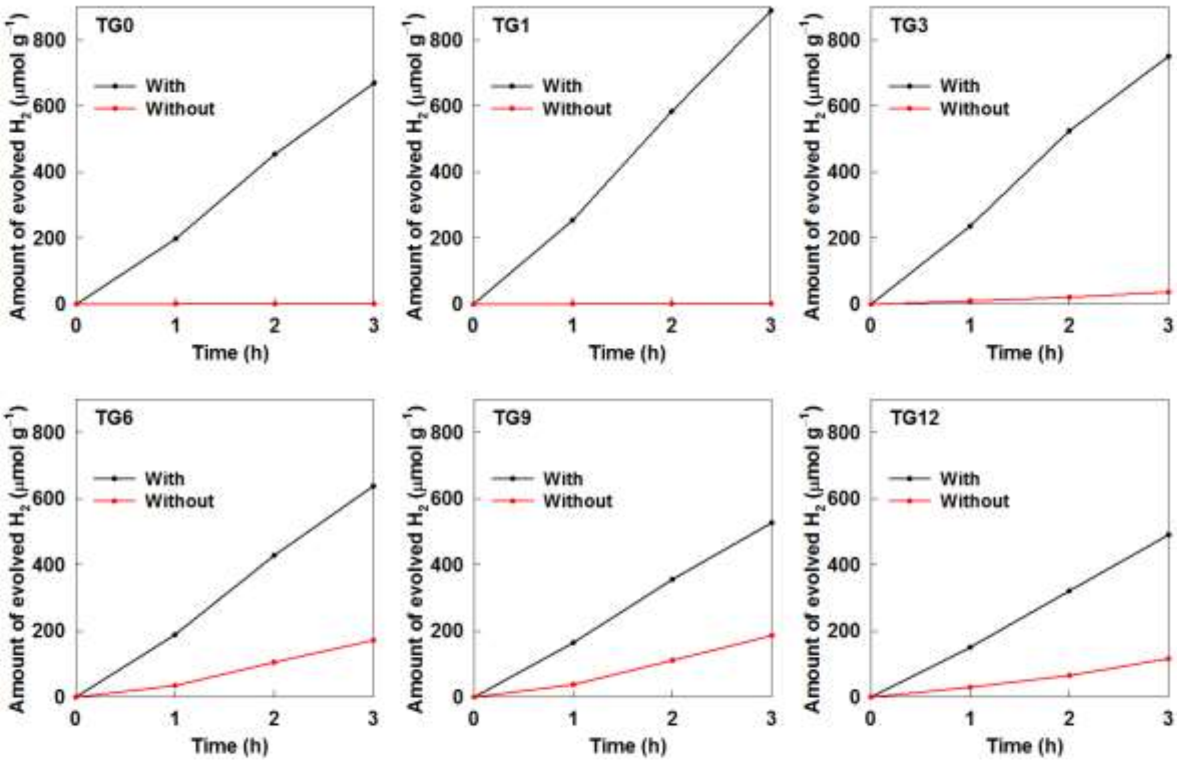

Figure S7. H_2 generation photocatalytic activities of TGn nanohybrids with/without hole scavenger.

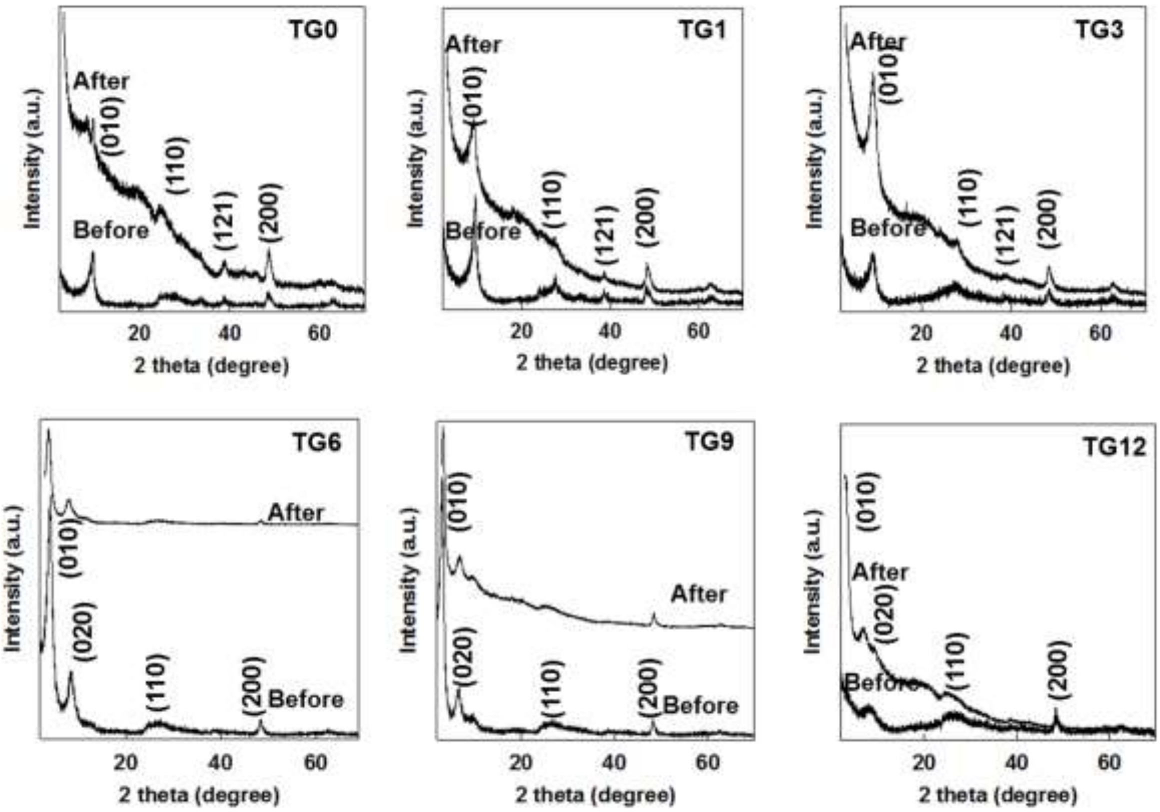

Figure S8. Powder XRD patterns of TGn nanohybrids before/after photocatalyst tests without hole scavenger.

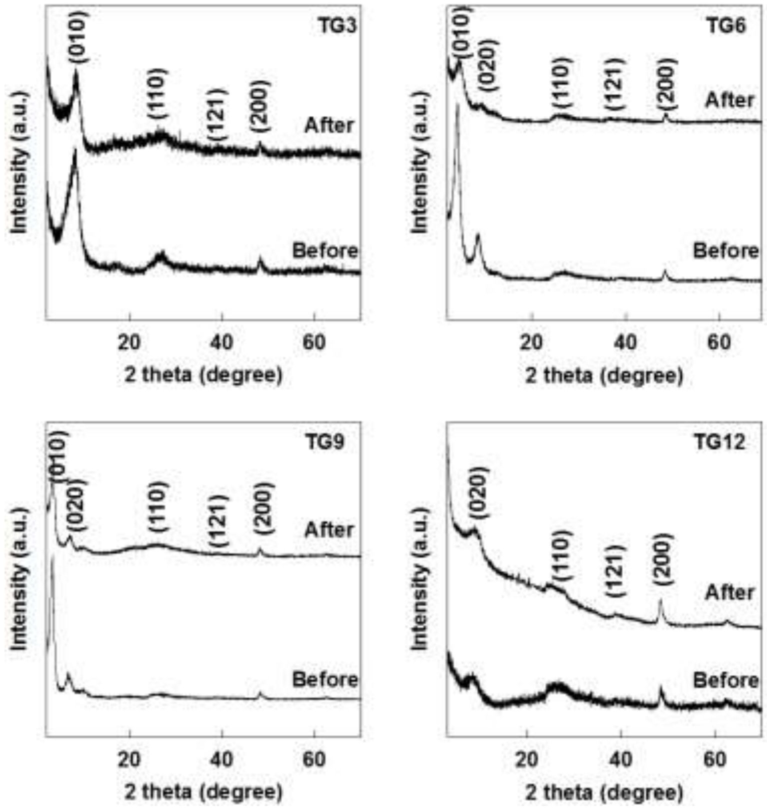

Figure S9. Powder XRD patterns of TGn nanohybrids before/after photocatalyst tests with hole scavenger.

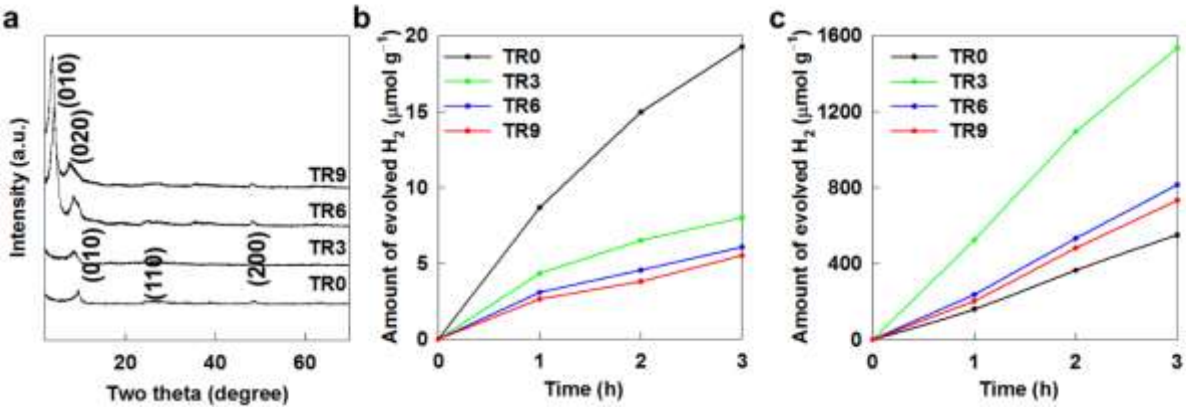

Figure S10. (a) Powder XRD patterns and photocatalytic activities for (b) visible light-induced H_2 evolution and (c) UV–visible-induced H_2 evolution by TRn nanohybrids.

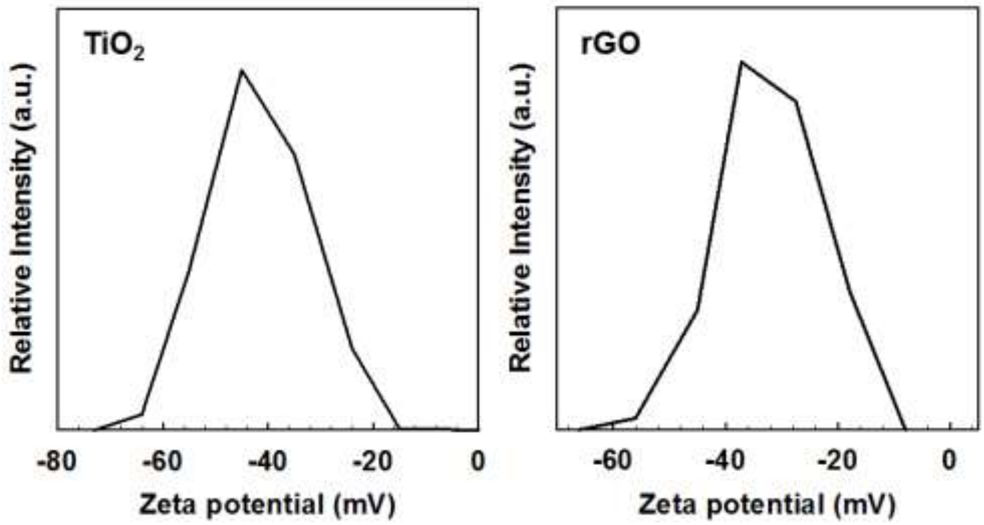

Figure S11. Zeta potential data of the colloidal suspensions of exfoliated TiO_2 nanosheet (NS) and exfoliated reduced graphene oxide (rGO) NS.

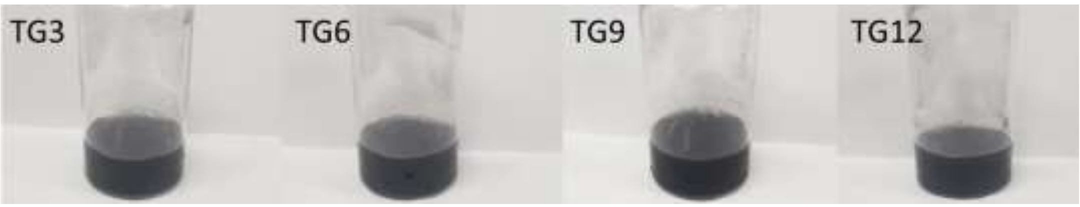

Figure S12. Dispersion ability of TGn nanohybrids for the photocatalyst tests.

Table S1. Relative concentrations of Ti^{3+} and Ti^{4+} ions for TGn nanohybrids.

Material	Ti^{3+}/Ti^{4+} ratio
H-TiO_2	0.11
TG12	0.12
TG9	0.17
TG6	0.22
TG3	0.25
TG0	0.33

Table S2. Ti K-edge extended X-ray absorption fine structure (EXAFS) fitting parameters for TGn nanohybrids.

Material	Bonding pair	Coordination number	R [Å]	σ^2 [10^{-3}×Å^2]
H-TiO_2	(Ti–O)	6	1.91	3.15
	(Ti–Ti)	2	2.62	4.67
	(Ti–Ti)	4	2.92	3.71
TG0	(Ti–O)	6	1.93	5.45
	(Ti–Ti)	2	2.63	5.35
	(Ti–Ti)	4	2.93	4.62
TG3	(Ti–O)	6	1.93	4.97
	(Ti–Ti)	2	2.63	7.13
	(Ti–Ti)	4	2.93	7.46
TG6	(Ti–O)	6	1.92	5.79
	(Ti–Ti)	2	2.62	7.30
	(Ti–Ti)	4	2.93	8.18
TG9	(Ti–O)	6	1.91	7.51
	(Ti–Ti)	2	2.62	9.67
	(Ti–Ti)	4	2.92	9.53
TG12	(Ti–O)	6	1.91	7.99
	(Ti–Ti)	2	2.62	9.74
	(Ti–Ti)	4	2.92	10.37

Table S3. Photoluminescence (PL) decay parameters of TGn nanohybrids.

Material	τ_1 [ns]	A_1 [%]	τ_2 [ns]	A_2 [%]	τ_3 [ns]	A_3 [%]
TG0	0.37	78.5	1.64	19.2	8.0	2.3
TG1	0.35	74.8	1.71	21.3	7.4	4.0
TG2	0.36	73.0	1.74	23.0	7.1	4.0
TG3	0.37	72.6	1.74	23.3	7.0	4.1
TG6	0.63	68.7	2.04	27.4	6.2	3.9
TG9	0.65	65.8	2.43	28.9	5.8	5.3
TG12	0.90	63.4	2.80	30.1	5.1	6.5
